# The occurrence and formation of monoterpenes in herbivore-damaged poplar roots

**DOI:** 10.1038/s41598-018-36302-6

**Published:** 2018-12-18

**Authors:** Nathalie D. Lackus, Sandra Lackner, Jonathan Gershenzon, Sybille B. Unsicker, Tobias G. Köllner

**Affiliations:** Max Planck Institute for Chemical Ecology, Department of Biochemistry, Hans-Knöll-Strasse 8, 07745 Jena, Germany

## Abstract

Volatiles are often released upon herbivory as plant defense compounds. While the formation of volatiles above-ground has been intensively studied, little is known about herbivore-induced root volatiles. Here, we show that cockchafer larvae-damaged roots of *Populus trichocarpa* and *P. nigra* release a mixture of monoterpenes, including (−)-α-pinene, (−)-camphene, (−)-β-pinene, *p*-cymene, and 1,8-cineole. Three terpene synthases, PtTPS16 and PtTPS21 from *P. trichocarpa* and PnTPS4 from *P. nigra*, could be identified and characterized *in vitro*. PnTPS4 was found to produce 1,8-cineole as sole product. PtTPS16 and PtTPS21, although highly similar to each other, showed different product specificities and produced γ-terpinene and a mixture of (−)-camphene, (−)-α-pinene, (−)-β-pinene, and (−)-limonene, respectively. Four active site residues were found to determine the different product specificities of the two enzymes. The expression profiles of *PtTPS16, PtTPS*2*1*, and *PnTPS4* in undamaged and herbivore-damaged poplar roots generally matched the emission pattern of monoterpenes, indicating that monoterpene emission in roots is mainly determined at the gene transcript level. Bioassays with *Phytophtora cactorum* (Oomycetes) revealed inhibitory effects of vapor-phase 1,8-cineole and (−)-β-pinene on the growth of this important plant pathogen. Thus herbivore-induced volatile monoterpenes may have a role in defense against pathogens that cause secondary infections after root wounding.

## Introduction

The production and emission of volatiles in response to herbivory is a well-studied phenomenon that has been described in a multitude of plant species. The released volatiles can fulfill different functions in direct and indirect plant defense, including the deterrence of herbivores and the attraction of herbivore enemies^1^. Moreover, such volatiles can act as signals in intra- and inter-plant communication where they warn other plant parts or neighboring plants against impending herbivore attacks^2,3^. Since herbivore-induced volatiles can be toxic to microorganisms, they have also been discussed as phytoanticipins, which may protect the wounding site by inhibiting secondary infections caused by phytopathogenic bacteria or fungi^1,4,5^.

While the formation and biological roles of volatiles emitted from herbivore-damaged above-ground organs have been intensively investigated during the last three decades, our knowledge about herbivore-induced root volatiles is still limited^6,7^. Maize (*Zea mays*) roots have been shown to release the sesquiterpene (*E*)-β-caryophyllene after damage by larvae of the root beetle *Diabrotica virgifera virgifera*^8,9^. (*E*)-β-Caryophyllene rapidly diffuses through the soil and can attract entomopathogenic nematodes able to attack and kill the beetle larvae. A mixture of four sesquiterpenes emitted from herbivore-damaged roots of Citrus trees (*Citrus paradise x Poncirus trifoliata*) was also shown to be attractive for entomopathogenic nematodes^10^. Herbivory of cockchafer larvae (*Melolontha melolontha*) on roots of apple trees (*Malus x domestica*) resulted in the emission of the monoterpene camphor^11^, while oak roots damaged by *M. hippocastani* larvae emitted a volatile mixture comprising 1,8-cineole, 1-octen-3-ol, octan-3-one, and the aromatic compound anisole^12^. Several *Brassica* species such as *B. nigra*, *B. juncea*, and *B. napus*, however, were shown to release mainly sulfur-containing volatiles derived from glucosinolate breakdown after infestation with the cabbage root fly (*Delia radicum*)^13,14^.

Herbivore-induced volatile blends are often dominated by mono- and sesquiterpenes, which are produced through the action of a specific class of enzymes called terpene synthases (TPS). Plant terpene synthases have mainly been described in seed plants^15^, but also in a few non-seed plants including lycophytes and mosses^16,17^. They catalyze a magnesium ion-dependent conversion of the ubiquitous precursors geranyl diphosphate (GPP), farnesyl diphosphate (FPP), and geranylgeranyl diphosphate (GGPP) into the different mono-, sesqui-, and diterpene skeletons, respectively, and thus determine the chemical nature of the formed terpenes^15^. Due to their reaction mechanisms, which include the formation of highly reactive carbocation intermediates, many terpene synthases have broader product specificity and produce mixtures of terpenes^15^. The exchange of single amino acids in the active site of TPS proteins often results in dramatic shifts in product specificity^15^. The opposite stereospecificity of two closely related maize terpene synthases TPS4 and TPS5, for example, is determined by only four amino acid differences in their active sites^18^.

During the last years, trees of the genus *Populus* have been established as model organisms to study the biochemical basis of terpene formation in trees. The genome of the western balsam poplar *Populus trichocarpa* contains 38 *TPS* gene models and about half of them have been characterized in previous studies^19–21^. Gene expression analysis and *in vitro* enzyme characterization revealed that the complex terpene blend emitted from gypsy moth caterpillar-damaged *P. trichocarpa* leaves can be fully explained by the enzyme activities of eight terpene synthases^19,20^. Four other TPS enzymes of *P. trichocarpa* could be characterized as diterpene synthases likely involved in the formation of non-volatile compounds in leaves and roots^21^. The biological relevance of the remaining 26 *TPS* genes in *P. trichocarpa*, however, is still unclear.

In the present study we performed volatile collection and gas chromatography-mass spectrometry analysis to identify and compare the volatile blends emitted from *M. melolontha*-damaged roots of the two poplar species *P. trichocarpa* and *P. nigra*. Heterologous expression of *TPS* genes in *Escherichia coli* and gene expression analysis using qRT-PCR allowed us to identify terpene synthases involved in monoterpene formation in *P. trichocarpa* and *P. nigra* roots. *In vitro* bioassays with the root pathogen *Phytophthora cactorum* and different volatile terpenes indicated that 1,8-cineole and (−)-β-pinene might be involved in plant defense against this generalist oomycete.

## Results

### Root herbivory leads to elevated monoterpene emission in poplar

As part of our ongoing research on volatile-mediated defenses in poplar, we analyzed the volatiles emitted from undamaged and *M. melolontha* (cockchafer)-damaged roots of *P. trichocarpa*, a species native to North America, and *P. nigra*, an indigenous European species. Trees were grown in sand-filled pots and a push-pull system was used to collect root volatiles from air pumped through the soil (Supplementary Fig. S1). Volatile analysis was conducted with gas chromatography-mass spectrometry (GC-MS). Root damage caused by the larvae was monitored after the experiment by measuring the total root biomass. For *P. trichocarpa*, cockchafer herbivory led to a significantly reduced root mass in comparison to undamaged controls (Supplementary Fig. S2). For *P. nigra*, however, we could not observe significant differences, although a slight trend of root biomass reduction in the grub treatments was visible (Supplementary Fig. S2).

Undamaged roots of both poplar species released considerable amounts of monoterpenes, including (−)-α-pinene, (−)-camphene, (−)-β-pinene, and 1,8-cineole (Table 1). Two further monoterpenes, *p*-cymene and an unidentified compound with a molecular mass of 136, could only be detected in the volatile bouquet of *P. trichocarpa*. Herbivory significantly increased the emission of 1,8-cineole from *P. trichocarpa*, while (−)-camphene and an unidentified monoterpene were significantly induced in herbivore-damaged roots of *P. nigra* (Table 1). Beside monoterpenes, both species also constitutively emitted the aromatic compounds benzaldehyde, benzyl alcohol, and salicylaldehyde, however, emission of these compounds was not influenced by the herbivore treatment (Supplementary Table S1). Considering that the plastics pots and the moist sand in the volatile collection system both might adsorb plant-released volatiles, our quantification of constitutive and herbivore-induced root volatiles in *P. trichocarpa* and *P. nigra* is likely an underestimation of the total volatile release.

**Table 1:** Emission of volatile monoterpenes from undamaged (ctr) and *Melolontha melolontha*-damaged (herb) roots of *Populus trichocarpa* and *P. nigra*. Emission levels are displayed as means ± SE in pg g^−1^ h^−1^ fresh weight (n = 8). *P*-values are based on the results from Student’s t-tests or from Mann-Whitney Rank Sum Tests between control and herbivore treatments. MT: monoterpene, NA: not detected.

	*P. trichocarpa*				*P. nigra*			
	ctr	herb	*P*-value	t-value	ctr	herb	*P*-value	t-value/ *T-value*
α-pinene	83.9 ± 34.3	149.3 ± 77.1	0.765	0.305	25.8 ± 9.2	78.1 ± 40.9	0.677	−0.426
camphene	52.6 ± 13.9	118.5 ± 31.6	0.061	−2.042	38.2 ± 11.8	666.4 ± 233.6	≤ 0.001	*37.00*
β-pinene	108.5 ± 27.9	207.9 ± 48.5	0.085	−1.854	31.3 ± 5.3	74.8 ± 21.9	0.058	−2.065
*p*-cymene	108.7 ± 31.9	200.6 ± 84.1	0.458	−0.764	NA	NA	NA	NA
1.8-cineole	258.4 ± 47.2	577.3 ± 150.0	0.048	2.168	101.3 ± 20.3	117.5 ± 22.7	0.578	−0.570
unidentified MT1	NA	NA	NA	NA	0.0 ± 0.0	20.1 ± 8.0	0.038	*48.00*
unidentified MT2	NA	NA	NA	NA	24.7 ± 3.6	30.5 ± 6.0	0.388	−0.892
unidentified MT3	NA	NA	NA	NA	82.9 ± 29.9	104.0 ± 33.9	0.625	−0.5

To measure the potential accumulation of volatile compounds in the roots, we extracted plant material with hexane and analyzed the extracts using GC-MS. While the aromatic volatiles benzaldehyde, benzyl alcohol, and salicylaldehyde accumulated in root material collected from *P. trichocarpa* and *P. nigra*, monoterpenes (camphene) could only be detected in the extracts of *P. nigra* (Supplementary Table S2). Interestingly, salicylaldehyde and benzaldehyde showed significantly increased accumulation after cockchafer herbivory, although their emission rates were not influenced by the treatment as already mentioned above (Supplementary Tables S1 and S2).

### Identification and characterization of three monoterpene synthases in *P. trichocarpa* and *P. nigra*

**Figure 1:**
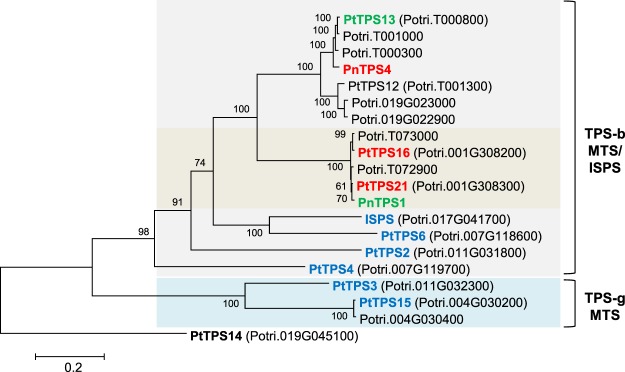
Dendrogram (maximum likelihood tree) of putative monoterpene synthases of *Populus trichocarpa* and *P. nigra* cloned in this study (red), their previously identified orthologues (green), other previously characterized *P. trichocarpa* TPS-b and TPS-g enzymes (blue), and further putative monoterpene synthases found in the *P. trichocarpa* genome version 3.0 (black). Bootstrap values (n = 1000) are shown next to each node. ISPS, isoprene synthase; MTS, monoterpene synthase; TPS-b and -g represent TPS subfamilies. Scale bar under the tree indicates number of substitutions per site.

The recently identified terpene synthase PnTPS1 produces camphene, α-pinene, β-pinene, and limonene and has been shown to be involved in herbivore-induced monoterpene formation in the leaves of *P. nigra*^22^. Another monoterpene synthase, the 1,8-cineole synthase PtTPS13, was reported to be expressed in herbivore-damaged leaves of *P. trichocarpa*^20^. Since products of both enzymes were found in the volatile bouquets of herbivore-induced *P. trichocarpa* and *P. nigra* roots (Table 1), we hypothesized that these or similar enzymes are also involved in the formation of root terpenes. To identify potential orthologues of *PnTPS1* and *PtTPS13* in *P. trichocarpa* and *P. nigra*, respectively, we amplified the open reading frames from cDNA made from *M. melolontha*-damaged root material. Amplification and cloning were successful and the resulting genes were designated *PtTPS21* (*P. trichocarpa* genome version 3.0 accession, Potri.001G308300) and *PnTPS4*, respectively, according to the poplar TPS nomenclature initiated in previous studies^19,20,22^. Sequencing of several amplicons revealed another *P. trichocarpa* gene with 97% nucleotide similarity to *PtTPS21* that was designated *PtTPS16* (Potri.001G308200). A sequence comparison and dendrogram analysis of predicted monoterpene synthase genes in the *P*. *trichocarpa* genome showed that *PtTPS16* and *PtTPS21* were part of a gene cluster comprising four members highly similar to each other (Fig. 1).

**Figure 2:**
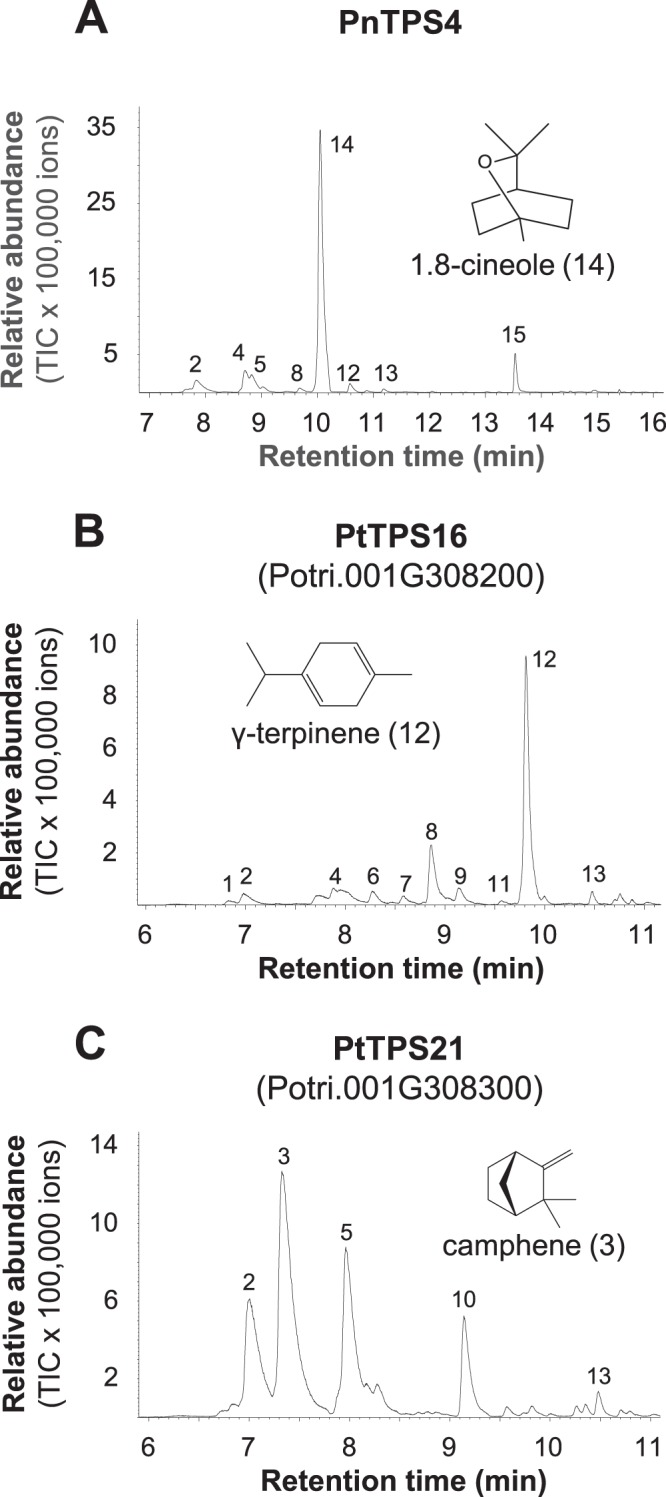
Biochemical characterization of the newly identified poplar root terpene synthases PnTPS4 (**A**), PtTPS16 (**B**), and PtTPS21 (**C**). The genes were heterologously expressed in *E. coli* and partially purified proteins were incubated with GPP as substrate. Enzyme products were analyzed using GC-MS. 1, α-thujene; 2, α-pinene*; 3, camphene*; 4, sabinene; 5, β-pinene*; 6, myrcene; 7, α-phellandrene; 8, α-terpinene*; 9, β-phellandrene; 10, limonene*; 11, ocimene; 12, γ-terpinene*; 13, terpinolene; 14, 1.8-cineole; 15, fenchyl alcohol. Compounds marked with * were identified by comparison of retention time and mass spectrum to those of authentic standards. Others were identified by database comparisons.

**Figure 3:**
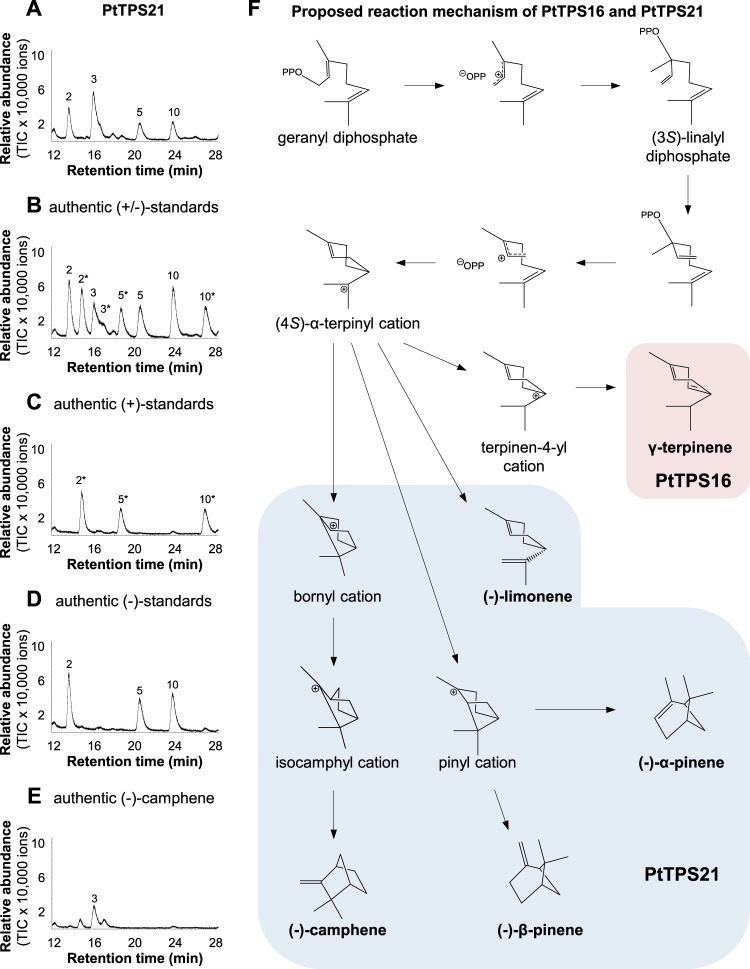
Stereochemical analysis of PtTPS21 enzyme products (**A–E**) and proposed reaction mechanism of PtTPS16 and PtTPS21 (**F**). 2, (−)-α-pinene; 3, (−)-camphene; 5, (−)-β-pinene; 10, (−)-limonene, 2*, (+)-α-pinene; 3*, (+)-camphene; 5*, (+)-β-pinene; 10*, (+)-limonene.

Heterologous expression of the N-terminal truncated open reading frames of the identified sequences lacking their putative signal peptides and subsequent enzyme assays confirmed monoterpene synthase activity for all tested enzymes. As expected, PtTPS21 and PnTPS4 had the same activity as their putative orthologues, PnTPS1 and PtTPS13, respectively, and produced camphene, α-pinene, β-pinene, and limonene (PtTPS21) and 1,8-cineole and a few minor products (PnTPS4) (Fig. 2). Interestingly, PtTPS16, although highly similar to PtTPS21, showed different product specificity and produced γ-terpinene together with a mixture of minor monoterpene products (Fig. 2). Chiral analysis of PtTPS21 enzyme products showed that all of the produced monoterpenes were exclusively formed as (−)-enantiomers (Fig. 3). When tested with the sesquiterpene precursor (*E,E*)-FPP, PnTPS4 showed activity and produced a mixture of sesquiterpens including (*E*)-α-bergamotene, (*E*)-β-farnesene, (*E,E*)-α-farnesene, sesquiphellandrene, (*Z*)-α-bisabolene, and nerolidol (Supplementary Fig. S3). However, since PnTPS4 was found to possess a signal peptide that targets the protein to the plastids (Supplementary Fig S4), it functions most likely as monoterpene synthase in planta. In contrast to PnTPS4, PtTPS16 and PtTPS21 were not active with (*E,E*)-FPP. The diterpene substrate (*E,E,E*)-GGPP was not accepted by the tested enzymes.

### Homology modeling of PtTPS16 and *in vitro* mutagenesis of active site residues

**Figure 4:**
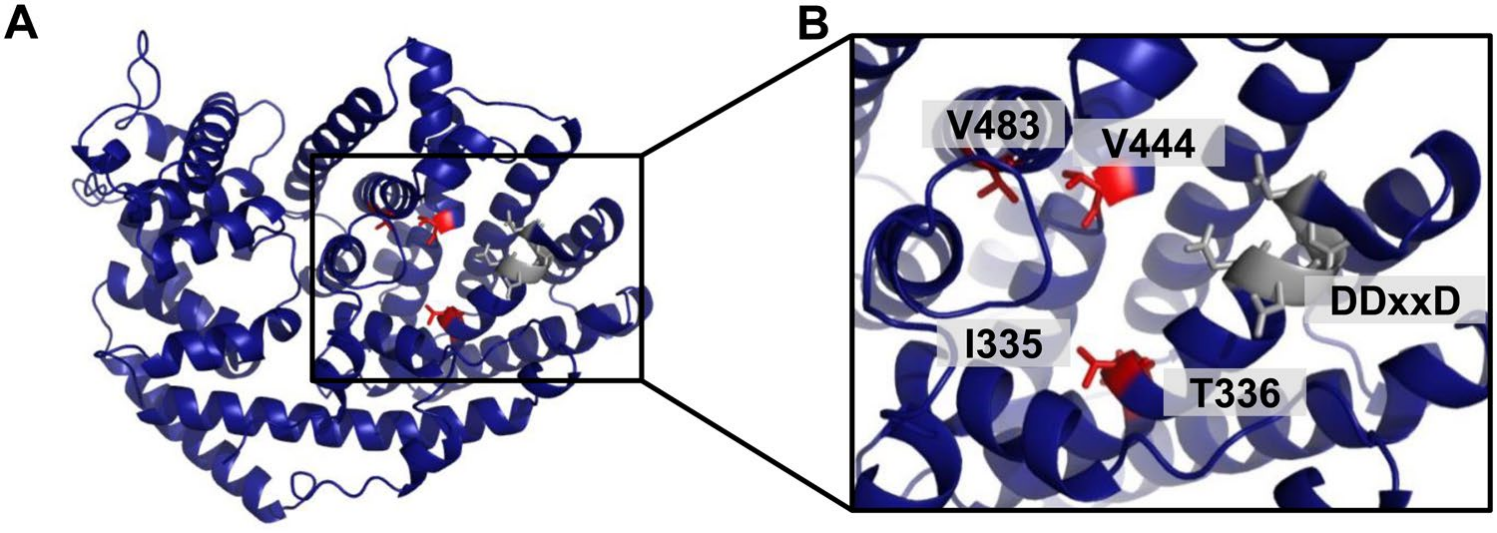
Structure model of PtTPS16. Models of N-terminal truncated PtTPS16 (**A**) and the active site of PtTPS16 (**B**) are shown. The conserved DDxxD motif is displayed in gray and the four amino acid residues that differ between the active sites of PtTPS16 and PtTPS21 are depicted in red.

**Figure 5:**
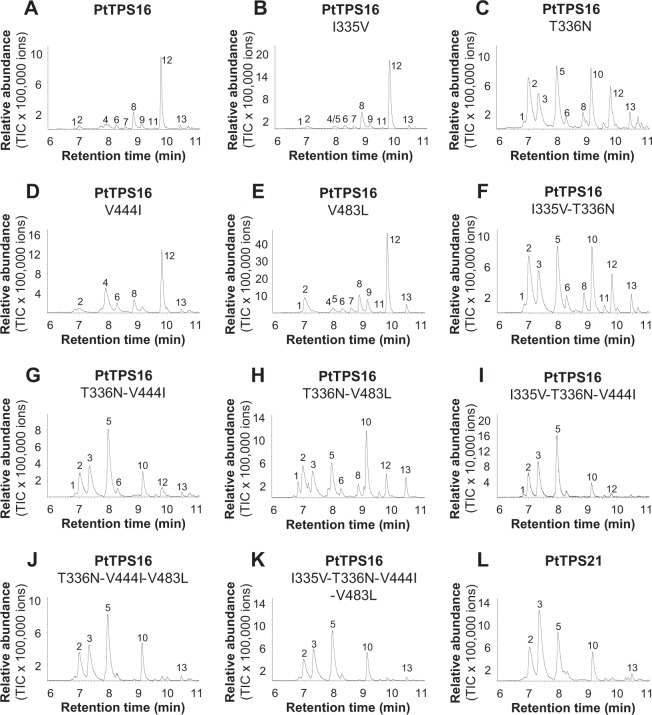
Biochemical characterization of PtTPS16 mutants generated using *in vitro* mutagenesis. GC-MS chromatograms representing the product spectra of wild type PtTPS16 (**A**), wild type PtTPS21 (**L**), and the different PtTPS16 mutants (**B**–**K**) are shown. Amino acid changes (one letter code) and their positions relative to the PtTPS16 sequence are indicated in the name of the mutants. The genes were heterologously expressed in *E. coli* and partially purified proteins were incubated with GPP as substrate. Enzyme products were analyzed using GC-MS. 1, α-thujene; 2, α-pinene*; 3, camphene*; 4, sabinene; 5, β-pinene*; 6, myrcene; 7, α-phellandrene; 8, α-terpinene*; 9, β-phellandrene; 10, limonene*; 11, ocimene; 12, γ-terpinene*; 13, terpinolene. Compounds marked with * were identified by comparison of retention time and mass spectrum to those of authentic standards. Others were identified by database comparisons.

To identify the amino acid residues that determine the observed differences in product specificity of PtTPS16 and PtTPS21, we performed homology modeling of the three-dimensional structure of PtTPS16 using the crystal structure of (+)-limonene synthase from *Citrus sinensis*^23^ as a template. Visualization of the resulting model and an amino acid sequence comparison of PtTPS16 and PtTPS21 revealed four amino acid substitutions within the active site pockets of the two proteins (Fig. 4; Supplementary Fig. S5). *In vitro* mutagenesis of the single residues isoleucine 335, valine 441, and valine 483 in PtTPS16 revealed no or only marginal changes in product specificity of the resulting mutant enzymes PtTPS16 I335V, PtTPS16 V441I, and PtTPS16 V483L, while a mutation of threonine 336 to asparagine led to an enzyme able to produce camphene, α-pinene, β-pinene, and limonene in addition to γ-terpinene (Fig. 5). Different combinations of single amino acid changes finally showed that the quadruple mutant PtTPS16 I335V, T336N, V441I, V483L had a product specificity highly similar to PtTPS21, although there were still minor quantitative differences in the product profiles of the mutant and PtTPS21 (Fig. 5).

### Gene expression analysis of terpene synthases in poplar roots

**Figure 6:**
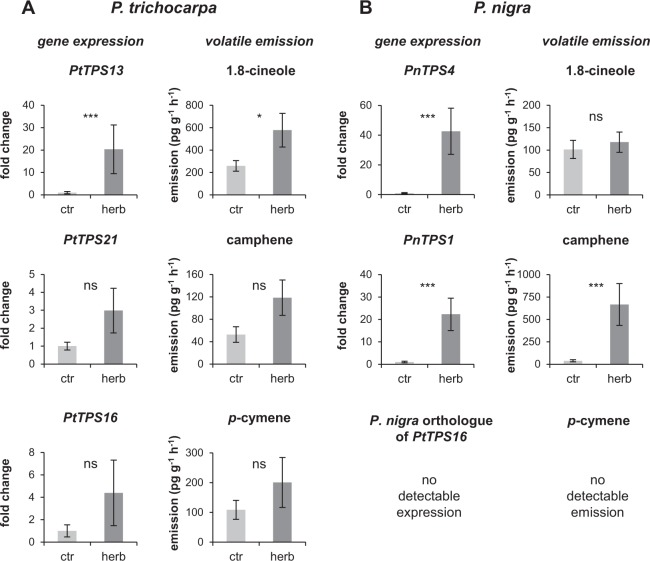
Expression of poplar root *TPS* genes and emission of major TPS products. Expression of *TPS* genes was analyzed using qRT-PCR. *TPS* expression and emission of monoterpenes are displayed for *M. melolontha*-damaged (herb) and undamaged (ctr) roots from *P. trichocarpa* and *P. nigra*. Means ± SE are shown (n = 8). Asterisks indicate statistical significance in Student’s t-tests or from Mann-Whitney Rank Sum Tests. *PtTPS13* (*P* ≤ 0.001, T = 36.00); *PtTPS21* (*P* = 0.209, T = 42.00); *PtTPS16* (*P* = 0.195, T = 55.00); *PnTPS4* (*P* ≤ 0.001, T = 36.00); *PnTPS1* (*P* ≤ 0.001, T = 36.00); *P. trichocarpa*: 1.8-cineole (*P* = 0.048, t = 2.168); camphene (*P* = 0.061, t = −2.042); *p*-cymene (*P* = 0.458, t = −0.764); *P. nigra*: 1.8-cineole (*P* = 0.578, t = −0.570); camphene (*P* ≤ 0.001, T = 37.00).

The expression levels of *P. trichocarpa PtTPS13*, *PtTPS16*, and *PtTPS21* and *P. nigra PnTPS4* and *PnTPS1* were measured in undamaged and *M. melolontha*-damaged roots using qRT-PCR. The 1,8-cineole synthase genes *PtTPS13* and *PnTPS4* were less expressed in undamaged roots than the other genes, but showed significant upregulation upon root herbivory (Fig. 6). Expression of *PnTPS1* was also significantly induced by the herbivore treatment. *PtTPS21* and *PtTPS16* showed a trend towards higher expression levels in damaged-roots, although it was not significant when compared to expression levels in undamaged control roots (Fig. 6).

### 1,8-Cineole reduces the growth of the plant pathogen *Phytophthora cactorum in vitro*

**Figure 7:**
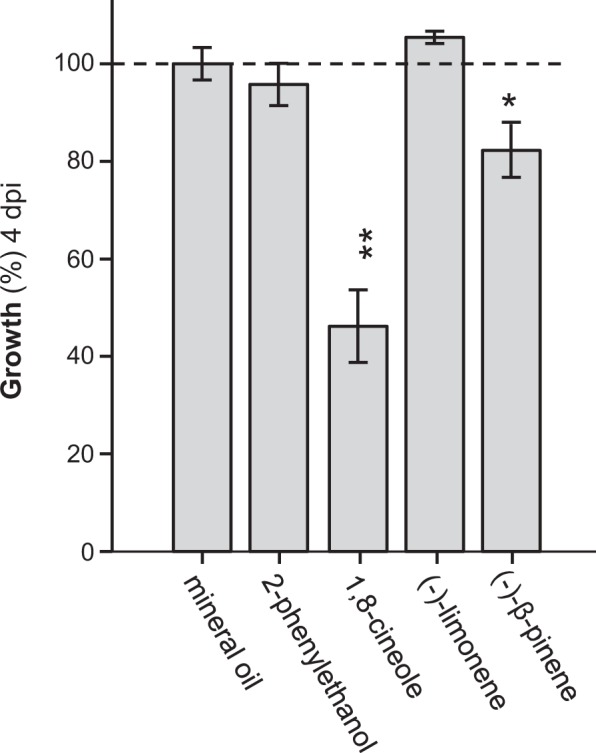
The effect of volatile monoterpenes on the growth of *Phytophthora cactorum* (Oomycetes). Mycelial growth of *P. cactorum* was measured in the presence of vapor-phase 2-phenylethanol, 1,8-cineole, (−)-limonene, and (−)-β-pinene. Pathogen growth in response to the individual compounds was compared to pathogen growth when exposed to pure mineral oil. The area of *P. cactorum* mycelium for the control treatment was set at 100% and growth under the influence of the different volatile compounds is shown relative to the mineral oil control. Means ± SE are shown (n = 5). Asterisks indicate significant differences (Student’s t-tests. 2-phenylethanol (*P* = 0.452, t = −0.797); 1,8-cineole (*P* = 0.001, t = −6.6); (−)-limonene (*P* = 0.166, t = 1.524); (−)-β-pinene (*P* = 0.023, t = −2.792)).

*Phytophthora cactorum* (Oomycetes) is a widespread plant pathogen that can infest numerous plant species including crops and trees^24^. Infection by this pathogen often results in root rot and causes massive yield losses or even plant death. To prove the hypothesis that herbivore-induced root monoterpenes might play a role in protecting wounded roots against soil-borne pathogens, we tested the influence of volatile 1,8-cineole, (−)-β-pinene, and (−)-limonene on the growth of *P. cactorum in vitro*. 2-Phenylethanol, a common plant volatile known to have antifungal activity^25^, was also included into the experiment. While 2-phenylethanol, (−)-limonene, and mineral oil as negative control had no influence on the growth of *P. cactorum*, 1,8-cineole and (−)-β-pinene significantly reduced the growth of this pathogen when present in the headspace (Fig. 7).

## Discussion

The emission of volatile organic compounds upon herbivory is part of a complex defense strategy that has been evolved as consequence of the ongoing evolutionary arms race between plants and their enemies. Both, the diverse biological roles of induced volatiles in plant defense as well as the biochemical basis of volatile formation have been intensively investigated in the past. However, the majority of this research focused on above-ground volatiles and thus our knowledge about root volatiles is still limited. Above-ground volatile blends are often dominated by mono- and sesquiterpenes that can fulfill diverse functions in plant-herbivore interactions^1^. Here we could show that undamaged and herbivore-damaged roots of the two poplar species *P. trichocarpa* and *P. nigra* produce and release a mixture of monoterpenes together with a few aromatic compounds (Table 1; Supplementary Table S1). These findings together with previous studies on maize, citrus, oak, and apple^8,10–12^ indicate that monoterpenes are also common, major components of herbivore-induced root volatile blends.

Interestingly, although emitted as volatiles, (−)-α-pinene, (−)-β-pinene, *p*-cymene, and 1,8-cineole could not be detected in root tissues of the investigated poplar trees. This suggests de novo biosynthesis, but also an efficient transport of the terpenes from the site of their formation to the rhizosphere that prevents any detectable accumulation in the plant. Root volatile emission without detectable tissue accumulation has also been reported in Arabidopsis, where the 1,8-cineole synthase AtTPS-Cin was found to be exclusively expressed in roots. While the enzyme product could not be detected in root extracts^26^, 1,8-cineole is indeed one of the major components of the Arabidopsis root volatile blend^27^. The 1,8-cineole synthase gene *AaTPS* in *Artemisia annua* is also expressed in roots but its product was not found in pentane extracts made from the respective tissues^28^. Although one cannot exclude a conversion of 1,8-cineole into another product, it is tempting to speculate that *A. annua* roots emit 1,8-cineole as shown for *Populus* and *Arabidopsis*.

Beside monoterpenes, poplar roots released considerable amounts of aromatic compounds including salicylaldehyde, benzaldehyde, and benzyl alcohol (Supplementary Table S1). In contrast to the volatile terpenes that showed no accumulation, the aromatic compounds accumulated in the roots (Supplementary Table S2). While their emission was not significantly influenced by the treatments, accumulation of salicylaldehyde and benzaldehyde was significantly increased upon herbivory. Thus one may hypothesize that both aldehydes are formed as degradation products of preformed salicinoids, a class of Salicaceae-specific phenolic glycosides derived from salicyl alcohol^29^. Or, the formation of these aromatic compounds might be independently induced by herbivory.

The 1,8-cineole synthase PtTPS13 and the camphene synthase PnTPS1 from *P. trichocarpa* and *P. nigra*, respectively, have recently been shown to be involved in herbivore-induced monoterpene formation in poplar leaves^20,22^. Since 1,8-cineole and camphene were also part of the poplar root volatile blend (Table 1), we tested the expression of *PtTPS13* and *PnTPS1* in undamaged and herbivore-damaged roots of *P. trichocarpa* and *P. nigra*. Moreover, we amplified and characterized the putative orthologues of *PtTPS13* and *PnTPS1*, *PnTPS4* and *PtTPS21*, respectively, and included them into gene expression analysis. The qRT-PCR experiment revealed that all analyzed genes were expressed in roots and showed significant induction (*PtTPS13, PnTPS4, PnTPS1*) or at least a trend for higher expression (*PtTPS21*) upon herbivory (Fig. 6). With one exception, the gene expression levels matched the emission of the respective major enzyme products, indicating that the terpene synthases contribute to monoterpene formation in *P. trichocarpa* and *P. nigra* roots. The 1,8-cineole synthase PnTPS4, however, was strongly upregulated upon herbivory, although 1,8-cineole was constitutively released from *P. nigra* roots (Fig. 6). This discrepancy might be explained by a conversion of 1,8-cineol to another terpenoid specifically upon herbivory in *P. nigra*. Interestingly, *P. trichocarpa* possesses two other *TPS* genes (Potri.T00100 and Potri.T000300) with high similarity to *PtTPS13* and *PnTPS4* (Fig. 1)^20^. Because amplification of Potri.T00100 and Potri.T000300 in *P. trichocarpa* failed, we speculate that they are not expressed in this species. However, their potential orthologues might be expressed in *P. nigra* roots and could be responsible for the constitutive 1,8-cineole emission observed from this organ.

Using primers specific for the (−)-camphene synthase *PtTPS21*, we amplified a further gene designated as *PtTPS16* from *P. trichocarpa* root cDNA. Despite a high sequence similarity of about 97% to PtTPS21, PtTPS16 had different product specificity and produced mainly γ-terpinene (Supplementary Fig. S3; Fig. 2). *PtTPS16* was found to be expressed in *P. trichocarpa* roots, but its product γ-terpinene could neither be detected in the root volatile blend nor in root extracts. Since γ-terpinene has been reported as a biosynthetic precursor for *p*-cymene in thyme (*Thymus vulgaris*)^30^ and *p*-cymene was found as one of the major root volatiles in *P. trichocarpa*, it is conceivable that *P. trichocarpa* also metabolizes γ-terpinene into this oxidized monoterpene. The absence of *p*-cymene in *P. nigra* could likely be explained by the loss of the respective *PtTPS16* orthologue or loss of its expression in this species (Fig. 6).

Terpene synthases catalyze complex reactions, which are usually initiated by the metal ion-mediated cleavage of the diphosphate group from the substrate. The resulting carbocation is highly reactive and can undergo a series of cyclizations and rearrangements such as hydride and methyl shifts. A final elimination of a proton or addition of water terminates the reaction (Fig. 3)^15^. Product specificity of terpene synthases is believed to be a consequence of the three-dimensional contour of the active site that restricts the conformations of the substrate and/or reactive cationic intermediates^31,32^. It has been shown that small structural changes in the active site caused by single amino acid mutations often dramatically alter the product specificity of terpene synthases (e.g.^21,33,34^). Indeed, when compared to each other, the active sites of PtTPS16 and PtTPS21 differed only in four amino acid residues (Fig. 4; Supplementary Fig. S5), and a series of site-directed mutagenesis experiments with PtTPS16 revealed that all of them are important for the interconversion of PtTPS16 into PtTPS21 (Fig. 5). However, the largest effect on product specificity was observed for the single mutant PtTPS16 T336N, suggesting that the threonine-asparagine polymorphism at position 336 in PtTPS16/21 mainly determines the different product outcome of the two enzymes. Notably, the product profiles of the PtTPS16 quadruple mutant and the PtTPS21 wild type were not completely identical but still showed minor quantitative differences. It is thus likely that amino acid residue substitutions near the active site also influence the backbone and/or side chain conformation of active site residues and thus contribute to the fine tuning of TPS product specificity. Such effects have already been observed in mutagenesis experiments conducted with the 5-*epi*-aristolochene synthase from tobacco^35,36^.

In general, wounding caused by herbivory or other mechanical stresses leads to favorable infection sites for microbial pathogens, providing them with nutrients and facilitates their entry into the plant tissue^37^. Many antimicrobial compounds have been described in plants, and among them are a variety of volatile mono- and sesquiterpenes^5^. Thus, we hypothesize that the increased emission of 1,8-cineole, (−)-camphene, and (−)-β-pinene from herbivore-damaged poplar roots is part of a defense reaction against soil-borne pathogens. Indeed, 1,8-cineole has been shown to have antimicrobial activities against a wide range of bacteria and fungi^38,39^. Moreover, its emission is induced by *Pseudomonas syringae* in Arabidopsis^27^. Our data showed that 1,8-cineole and (−)-β-pinene are also toxic for an oomycete root pathogen. Bioassays with *P. cactorum*, a common pathogen with a wide host range including many tree species, showed significantly reduced growth when it was cultivated in the presence of vapor-phase 1,8-cineole or (−)-β-pinene (Fig. 7). Other tested volatiles such as 2-phenylethanol and (−)-limonene, however, had no effect on *P. cactorum* growth, although they have been described as antimicrobial compounds in previous studies^25,39^. Our data suggest specific roles of single monoterpenes in different root-pathogen interactions.

Beside a potential function in pathogen defense, poplar root monoterpenes also might directly influence insect attackers. 1,8-Cineole and limonene, for example, have been shown to have insecticidal activity against the American wheat weevil *Rhyzopertha dominica* and the red flour beetle *Tribolium castaneum*^40^ and it is conceivable that they are toxic for *M. melolontha* and other beetles as well. Moreover, root volatiles may influence the behavior of the beetle larvae. In bioassays with pure compounds, Eilers could show that γ-terpinene and benzaldehyde were both repellent for *M. melolontha* larvae while camphene and α-pinene attracted the larvae^41^. Because benzaldehyde, camphene, and α-pinene are all present in the root volatile blend of poplar, the combined effect of the blend on the beetle larvae is unclear. How the behavior of *M. melolontha* is influenced by the complete poplar volatile blend and whether there are potential synergistic effects among single root volatiles should be addressed in future studies.

Recent research showed that root volatiles play also roles in indirect plant defense. Herbivore-damaged roots of maize and citrus trees, for example, release volatile sesquiterpenes that attract entomopathogenic nematodes able to infest the attacking beetle larvae^8,10^. Whether herbivore-induced poplar monoterpenes can attract nematodes is still unclear. However, it has been shown that infection of *M. melolontha* larvae with nematodes of the genera *Heterorhabditis* and *Steinernema* increased larval mortality^42^, and thus the attraction of nematodes by poplar root volatiles might provide an efficient defense against herbivorous beetles.

## Material and Methods

### Plants and insects

Western balsam poplar (*Populus trichocarpa*, clone Muhle-Larsen, P&P Baumschule, Eitelborn, Germany) and black poplar (*P. nigra*, clone f41)^43^ trees were propagated from monoclonal stem cuttings and grown under summer conditions in the greenhouse (24 °C, 60% rel. humidity, 16 h/8 h light/dark cycle) in sand (Klasmann-Deilmann, Geeste, Germany), until they reached about 0.5 m in height.

Cockchafer larvae (*Melolontha melolontha*) were collected from meadows near Mespelbrunn/Spessart (Germany). Insects were reared individually in 200 ml plastic beakers filled with a mix of potting soil and grated carrots in a wine cooler operating in the dark at 12 °C.

### Volatile collection

For volatile measurements, pots with single trees were completely enclosed in PET bags (“Bratschlauch”, Toppits, Minden, Germany) by fixing one end of the bags to the stem of the tree with a cable binder and closing the other end also with a cable binder. Volatiles were collected using a dynamic push-pull system as shown in Supplementary Fig S1. Air flow was maintained in the system through teflon tubes. Charcoal filtered air was pumped into the bags at a flow of 1 l min^−1^. A portion of the aspirated air (flow: 0.6 l min^−1^) was withdrawn with a 2nd pump and passed through a cooling trap (a glass bottle that was cooled down to 9 °C) to remove air humidity. A filter packed with 30 mg Poropak (ARS, Inc., Gainsville, USA) was used to adsorb the volatile compounds. Volatiles were collected for 68 h. After the collection, the volatile compounds were desorbed by eluting the filter with 200 µl dichloromethane containing nonyl acetate as an internal standard (10 ng µl^−1^). Samples were stored at −20 °C until gas chromatography analysis. During the experiment, all plants were watered daily by injecting 30 ml of tap water into the sand through a tiny hole in the plastic bag that was tightly closed after watering.

For the herbivore treatment, two *M. melolontha* larvae (3d instar) were buried in each pot. Larvae were allowed to feed throughout the duration of the experiment. Volatile collections of sand-filled pots without trees and volatile collections of *M. melolontha* larvae were performed as negative controls. Root material was harvested and weighed immediately at the end of the volatile collection, flash-frozen with liquid nitrogen and stored at −80 °C until further sample preparation.

### Hexane extraction of root tissue

To determine terpene accumulation in poplar roots, 100 mg of root powder was extracted in a GC glass vial with 400 µl hexane including 10 ng/uL nonyl acetate as internal standard. The extracts were shaken for one hour at 300 rpm and incubated over night at room temperature. After centrifugation for 10 min at 5,000 x g, the supernatant was analyzed using GC.

### RNA extraction and reverse transcription

Total RNA was isolated from ground plant tissue using an InviTrap Spin Plant RNA kit (Stratec, Berlin, Germany) according to manufacturer’s instructions. RNA concentration and purity were assessed using a spectrophotometer (NanoDrop 2000c, Thermo Scientific, Wilmington, DE, USA). RNA was treated with DNaseI (ThermoFisher Scientific, https://www.thermofisher.com) prior to cDNA synthesis. Single-stranded cDNA was prepared from 1 µg of DNase-treated RNA using SuperScriptTM III reverse transcriptase and oligo (dT12–18) primers (Invitrogen, Carlsbad, CA, USA).

### Isolation of TPS genes

Open reading frames (ORF) encoding the N-terminal truncated versions of PtTPS16 (Δ42), PtTPS21 (Δ42), and PnTPS4 (Δ42) lacking the putative signal peptides predicted with the programs ChloroP (http://www.cbs.dtu.dk/services/ChloroP/) and TargetP (http://www.cbs.dtu.dk/services/TargetP/) (Supplementary Fig. S4) were amplified from cDNA made from herbivore-damaged root material with the primers listed in Supplementary Table S3. The PCR products obtained were inserted into the expression vector pET100/D-TOPO^®^ (ThermoFisher Scientific, https://www.thermofisher.com) (*PtTPS16* and *PtTPS21*) or pASK-IBA7 (IBA-GmbH, Göttingen, Germany) (*PnTPS4*) and the cloned genes were fully sequenced.

### *In vitro* mutagenesis

For site-directed mutagenesis, 100 ng pET100/D-TOPO® vector containing the N-terminal truncated ORF of *PtTPS16* was used as template in a mutagenesis PCR (18 cycles, Phusion® High-Fidelity DNA Polymerase (ThermoFisher Scientific), according to manufacturer’s instructions. The primers used contained the desired mutations and their sequences are given in Supplementary Table S3. After PCR, the plasmid template DNA was digested with *Dpn*I and the reaction mixture was inserted and amplified in *E. coli* TOP10 (Invitrogen). The mutagenized constructs were fully sequenced before expression.

### Heterologous expression and TPS enzyme assays

The *E. coli* strain BL21 Star™ (DE3) (ThermoFisher Scientific) was used for expression of *PtTPS16/21* while *PnTPS4* was expressed in *E. coli* TOP10 (Invitrogen). Cultures were grown at 37 °C, induced at an OD_600_ = 0.6 with 1 mM IPTG (*PtTPS16/21*) or 200 ug l^−1^ anhydrotetracycline (*PnTPS4*) and subsequently placed at 18 °C and grown for another 20 hours. The cells were collected by centrifugation and disrupted by a 4 × 20 s treatment with a sonicator (Bandelin UW2070, Berlin, Germany) in chilled extraction buffer (10 mM Tris-HCl (pH 7.5), 1 mM dithiothreitol, 10% (v/v) glycerol). Cell fragments were removed by centrifugation at 14,000 g and the supernatant was used for enzyme assays.

To determine the catalytic activity of the different terpene synthases, enzyme assays were performed in a Teflon-sealed, screw-capped 1 ml GC glass vial containing 40 μl of the bacterial extract and 60 µl assay buffer with 10 μM substrate (GPP or (*E,E*)-FPP) and 10 mM MgCl_2_. A solid phase microextraction (SPME) fiber consisting of 100 µm polydimethylsiloxane (SUPELCO, Belafonte, PA, USA) was placed into the headspace of the vial for 60 min incubation at 30 °C. For analysis of the adsorbed reaction products, the SPME fiber was directly inserted into the injector of the gas chromatograph (see below). Potential diterpene synthase activity was tested in assays with 50 µM (*E,E,E*)-GGPP as substrate. Assays were overlaid with 100 µl hexane and incubated for 60 minutes at 30 °C. Two microliters of the hexane phase were injected into the gas chromatograph. To determine the chirality of the produced monoterpenes, assays were set up as described above, containing 50 µM GPP as substrate, and overlaid with 100 µl hexane. After incubation for 60 min at 30 °C, the hexane phase was collected and analyzed using chiral gas chromatography-mass spectrometry (GC-MS).

### GC-MS analysis of volatiles and enzyme products

Qualitative and quantitative analysis of root volatiles and terpene accumulation was conducted using an Agilent 6890 Series gas chromatograph coupled to an Agilent 5973 quadrupole mass selective detector (interface temp, 250 °C; quadrupole temp, 150 °C; source temp, 230 °C; electron energy, 70 eV) or a flame ionization detector (FID) operated at 300 °C, respectively. The constituents of the volatile bouquet were separated using a ZB5 column (Phenomenex, Aschaffenburg, Germany, 30 m × 0.25 mm × 0.25 µm) and He (MS) or H_2_ (FID) as carrier gas. The sample (2 µL) was injected without split at an initial oven temperature of 45 °C. The temperature was held for 2 min and then increased to 180 °C with a gradient of 6 °C min^−1^, and then further increased to 300 °C with a gradient of 60 °C min^−1^ and a hold of 2 min. Compounds were identified by comparison of retention times and mass spectra to those of authentic standards obtained from Fluka (Seelze, Germany) and Sigma-Aldrich (St. Louis, MO, USA), or by reference spectra in the Wiley and National Institute of Standards and Technology libraries.

TPS enzyme products were analyzed and identified using GC-MS as described above for poplar root volatiles. The sample (SPME) was injected without split at an initial oven temperature of 45 °C. The temperature was held for 2 min, then increased to 130 °C with a gradient of 7 °C min^−1^, and further increased to 300 °C with a gradient of 100 °C min^−1^ and a hold of 2 min.

Chiral GC-MS analysis was performed using a Rt™-βDEXsm-column (Restek, Bad Homburg, Germany). The temperature was first held at 45 °C for 28 min and then increased to 200 °C with a gradient of 100 °C min^−1^ and a hold for 2 min. Enantiomers were identified using authentic standards obtained from Fluka, Sigma-Aldrich, and Merck (Darmstadt, Germany).

### Gene expression analysis (qRT-PCR)

cDNA was prepared as described above and diluted 1:10 with water. qPCR primers for poplar *TPS* genes were designed having a Tm ≥ 60 °C, a GC content between 50–58%, and a primer length of 22–24 nucleotides (Supplementary Table S3). The amplicon size was between 110 to 140 base pairs. Expression analysis of *PtTPS13* was performed with primers previously published^20^. The specificity and efficiency of the primers were confirmed by agarose gel electrophoresis, melting curve analysis, standard curve analysis, and by sequence verification of cloned PCR amplicons. *Ubiquitin* was used as a reference gene^44^. The expression of *PtTPS13* was analyzed using Brilliant III Ultra-Fast SYBR^®^ Green QPCR Master Mix (Stratagene, Carlsbad, CA, USA) with the following PCR conditions: Initial incubation at 95 °C for 3 min followed by 40 cycles of amplification (95 °C for 5 sec, 60 °C for 10 sec). Expression of *PtTPS16*, *PtTPS21*, *PnTPS1*, and *PnTPS4* was analyzed using HiDi DNA Polymerase (Genaxxon Bioscience GmbH, Ulm, Germany) and Green DNA Dye (Genaxxon Bioscience GmbH) as dye using the following PCR conditions: Initial incubation at 95 °C for 3 min followed by 40 cycles of amplification (95 °C for 15 sec, 60 °C for 10 sec, 72 °C for 30 sec). For all measurements, plate reads were taken at the end of the extension step of each cycle and data for the melting curves were recorded at the end of cycling from 60 °C to 95 °C. All samples were run on the same Bio-Rad CFX Connect™ Real-Time PCR Detection System (Bio-Rad Laboratory, Hercules, CA, USA) in an optical 96-well plate. Eight biological replicates were analyzed in triplicate.

### *Phytophthora cactorum* cultivation and bioassays

*Phytophthora cactorum* (Oomycetes) was obtained from the Leibniz Institut DSMZ-German Collection of Microorganisms and Cell Cultures GmbH (Braunschweig, Germany). The generalist root pathogen was grown *via* mycelial inoculation in Petri dishes containing tomato juice medium. A 1.5 l quantity of medium contained 300 ml tomato juice (“Bio” quality from Netto supermarket), 4.5 g CaCO_3_ (Roth, Karlsruhe, Germany), and 11.25 g agar-agar, filled to full volume with triple distilled water (adjusted to pH 7.2) at room temperature.

For bioassays, the inoculum was punched out with a cork borer (4 mm diameter) and placed at the center of fresh Petri dishes containing 25 ml of tomato juice medium. Small PCR tubes containing either 20 µl of 1,8-cineole (Sigma-Aldrich), (−)-limonene (Sigma-Aldrich), (−)-β-pinene (Sigma-Aldrich), 2-phenylethanol (Sigma-Aldrich), or mineral oil were placed on the very edge of the Petri dishes. Petri dishes were sealed and stored in an incubator at room temperature. Pathogen growth was monitored daily by taking a digital photograph that was later analyzed using Adobe Photoshop CS5 (Adobe Systems, San Jose, CA, USA). Mycelial growth in the control treatment, where the pathogen was just exposed to mineral oil in the PCR tubes, was set at 100% and growth under the influence of the different volatile compounds is shown relative to the control. The fourth replicate of the *2*-phenylethanol treatment was contaminated and was therefore not considered for analyses.

### Sequence analysis and phylogenetic tree construction

An alignment of all *P. trichocarpa TPS-g* and *TPS-b* genes and characterized *TPS* genes from *P. nigra* was constructed using the MUSCLE (codon) algorithm (gap open, −2.9; gap extend, 0; hydrophobicity multiplier, 1.2; clustering method, UPGMB) implemented in MEGA6^45^. Tree reconstruction was done with MEGA6 using a maximum likelihood algorithm (model/method, Tamura 3-paramter model; substitutions type, nucleotide; rates among sites, gamma distributed with invariant sites (G + I); gamma parameters, 5; gaps/missing data treatment, partial deletion; site coverage cutoff, 95%). A bootstrap resampling analysis with 1000 replicates was performed to evaluate the tree topology.

### TPS structure modeling

A model of the three dimensional structure of PtTPS16 was generated using the Swiss-Model Server (https://swissmodel.expasy.org/). For modeling, the poplar TPS sequence was fitted to the template structure of (+)-limonene synthase from *Citrus sinensis*^23^. The resulting model was visualized with the program Swiss-PdbViewer3.7 (https://spdbv.vital-it.ch/).

### Statistical analysis

Throughout the manuscript, data are presented as means ± SE. To compare volatile emissions, expression of *TPS*, and loss of root biomass in undamaged and herbivore-damaged *P. trichocarpa* and *P. nigra* roots, Student’s t-tests or Mann-Whitney Rank Sum Tests were performed with SigmaPlot 11.0 for Windows (Systat Software Inc., https://systatsoftware.com). Whenever necessary, the data were log transformed to meet statistical assumptions such as normality and homogeneity of variances. For statistical analysis of *P. cactorum* bioassays, a student’s t-test was carried out in SPSS 20 (IBM, Armonk, NY, USA). Normality distribution and heterogeneity of variances was tested.

### Accession numbers

Sequence data for genes in this article can be found in the GenBank under the following identifiers: *PtTPS16* (MH541838), *PtTPS21* (MH541839), *PnTPS4* (MH541837).

## Electronic supplementary material

Supplemental figures and tables

## Data Availability

All data generated or analyzed during this study are included in the main text or supplement of this published article.
