# Supplementary material for: The occurrence and formation of monoterpenes in herbivore-damaged poplar roots
Source: Sci Rep. 2018 Dec 18;8:17936. doi: 10.1038/s41598-018-36302-6 (PMC6299004; doi:10.1038/s41598-018-36302-6)
Supplement: Supplementary file 1 — Supplemental figures and tables [file 41598_2018_36302_MOESM1_ESM.pdf]

Supplementary material:

# The occurrence and formation of monoterpenes in herbivore-damaged poplar roots

Nathalie D. Lackus, Sandra Lackner, Jonathan Gershenzon, Sybille B. Unsicker and Tobias G. Köllner*

Max Planck Institute for Chemical Ecology, Department of Biochemistry, Hans-Knöll-Strasse 8, 07745 Jena, Germany

*Corresponding author. Tel.: +49 (0) 3641 57 1329; fax: +49 (0) 3641 57 1302. E-mail address: koellner@ice.mpg.de.

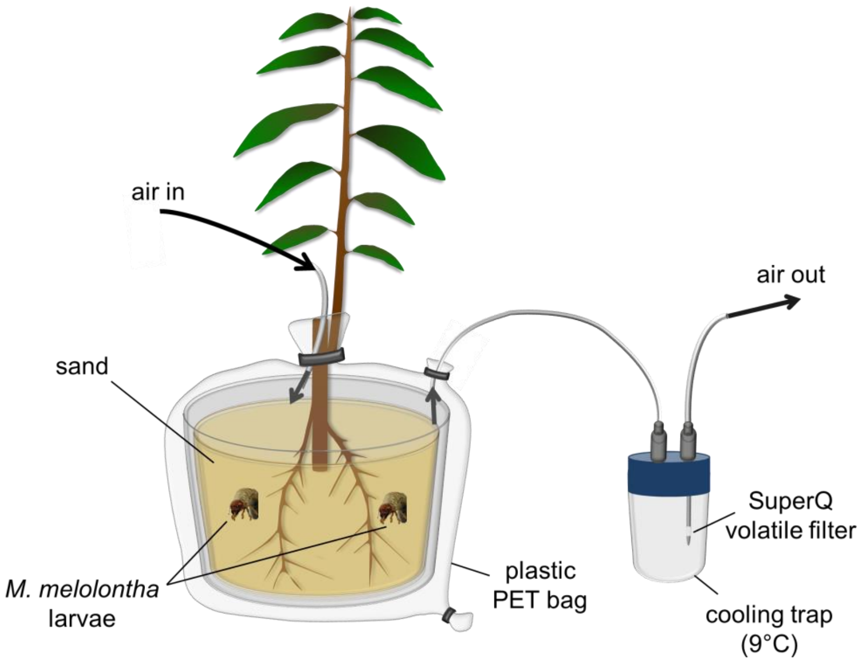


**Supplementary Figure S1: Root volatile collection system.** Volatiles were sampled for 68 h with a dynamic push-pull system and a trap packed with Poropak adsorbent. Trees were grown in sand and the pots were bagged in PET foil for volatile collection.

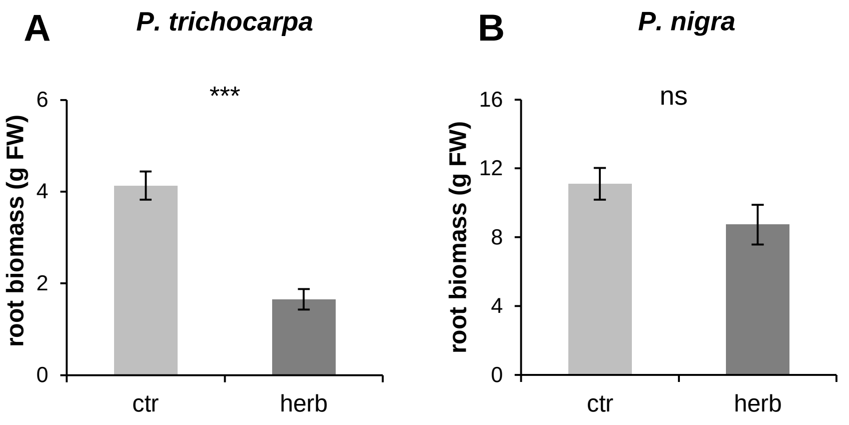


**Supplementary Figure S2: Effect of herbivory on root mass of *Populus trichocarpa* (A) *and P. nigra* (B) trees.** Root masses are displayed for undamaged roots (ctr) and roots damaged by cockchafer (*Melolontha melolontha*) larvae (herb) and shown as means ± SE in g (n = 8). Asterisks indicate statistical significance in Student's t-tests. *P. trichocarpa* (*P* ≤ 0.001 , t = 6.973); *P. nigra* (*P* = 0.108, t = 1.717).

**PnTPS4**

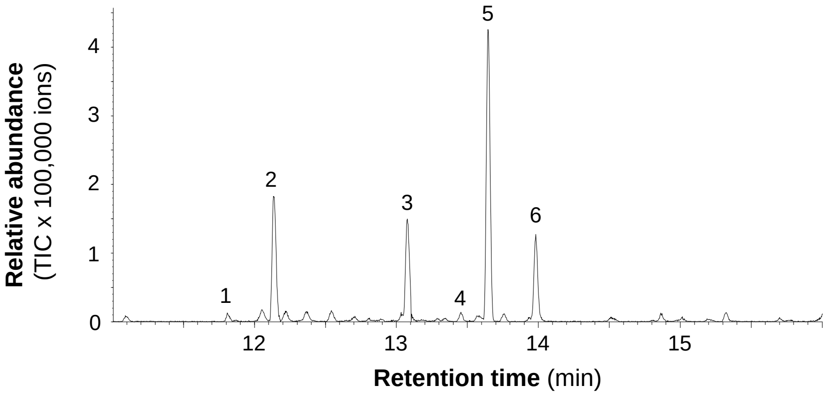


**Supplementary Figure S3: Sesquiterpene synthase activity of PnTPS4.** The gene was heterologously expressed in *E. coli* and partially purified protein was incubated with (*E,E*)-FPP as substrate. Enzyme products were analyzed using GC-MS. 1, (*E*)-α-bergamotene; 2, (*E*)-β-farnesene; 3, (*E,E*)-α-farnesene; 4, sesquiphellandrene; 5, (*Z*)-α-bisabolene; 6, nerolidol. Compounds were tentatively identified by database comparisons.

**A**

```
### chlorop v1.1 prediction results ###########################
Number of query sequences: 3

Name                    Length     Score    cTP    CS-       cTP-
                                                   score     length
---------------------------------------------------------------
PnTPS4_full_length        589      0.568    Yes    11.435      40
PtTPS16_full_length       593      0.569    Yes    11.435      38
PtTPS21_full_length       593      0.570    Yes    11.435      38
---------------------------------------------------------------
```

**B**

```
### targetp v1.1 prediction results ##################################
Number of query sequences:  3
Cleavage site predictions not included.
Using PLANT networks.

Name                   Len     cTP    mTP     SP  other  Loc  RC
----------------------------------------------------------------------
PnTPS4_full_length     589   0.913  0.108  0.083  0.040   C    1
PtTPS16_full_length    593   0.826  0.486  0.029  0.016   C    4
PtTPS21_full_length    593   0.894  0.352  0.028  0.025   C    3
----------------------------------------------------------------------
cutoff                       0.000  0.000  0.000  0.000
```

**Supplementary Figure S4: Signal peptide prediction of PnTPS4, PtTPS16, and PtTPS21.** Signal peptide (cTP) prediction was done using the web-based prediction programs chlorop v1.1 (http://www.cbs.dtu.dk/services/ChloroP/) (A) and targetp v1.1 (http://www.cbs.dtu.dk/services/TargetP/) (B).

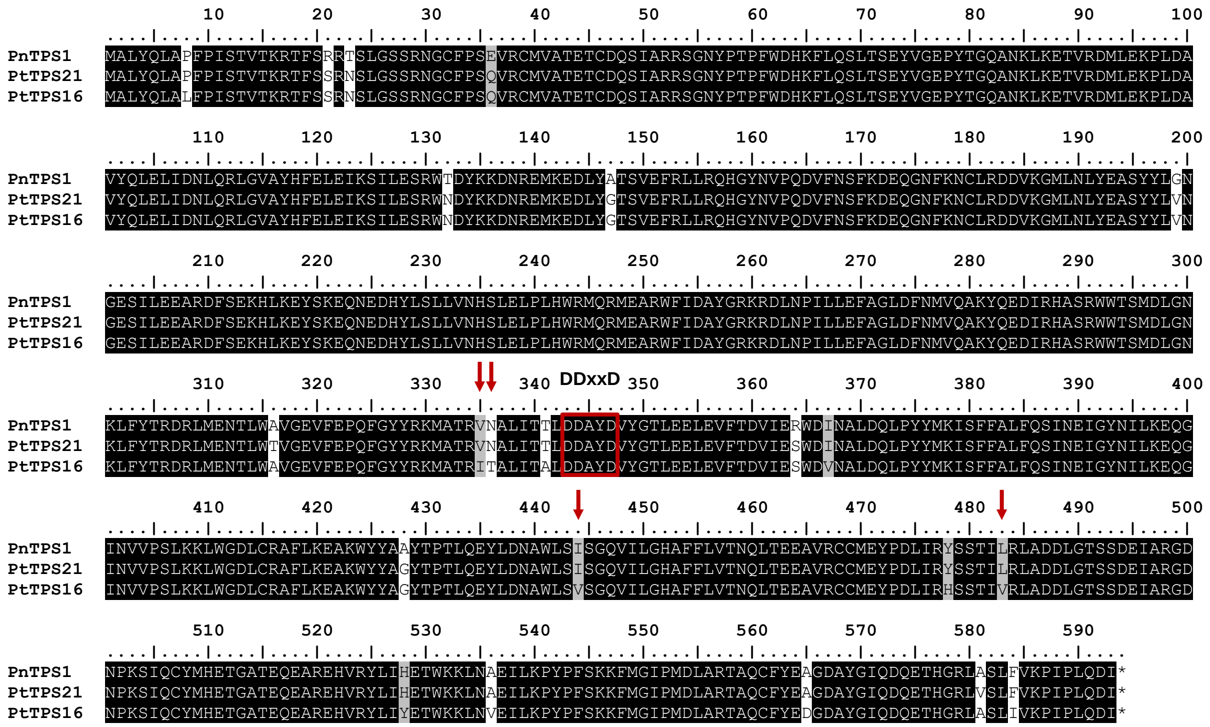


**Supplementary Figure S5: Amino acid sequence comparison of PtTPS16 and PtTPS21 from *Populus trichocarpa* and PnTPS1 from *P. nigra*.** Amino acids with identical side chains are marked by black boxes and amino acids with similar side chains are marked by gray boxes. Red arrows highlight different amino acid residues in the active site. The conserved DDxxD motif is marked in red.

**Supplementary Table S1.** Emission of aromatic volatile compounds from undamaged (ctr) and *Melolontha melolontha*-damaged (herb) roots of *Populus trichocarpa* and *P. nigra.* Volatiles were analyzed using GC-MS/FID and emission levels are displayed as means ± SE in pg $g^{-1}$ $h^{-1}$ fresh weight (n = 8). *P*-values are based on the results from Student's t-tests or from Mann-Whitney Rank Sum Tests between control and herbivore treatments.

| | ***P. trichocarpa*** | | | | ***P. nigra*** | | | |
|---|---|---|---|---|---|---|---|---|
| | **ctr** | **herb** | ***P*-value** | **t-value** | **ctr** | **herb** | ***P*-value** | **t-value/ *T-value*** |
| benzaldehyde | 123 ± 44 | 297 ± 98 | 0.063 | -2.023 | 69 ± 15 | 75 ± 34 | 0.442 | *76.00* |
| benzyl alcohol | 244 ± 63 | 269 ± 61 | 0.765 | -0.305 | 284 ± 68 | 364 ± 131 | 0.931 | 0.0887 |
| salicylaldehyde | 749 ± 401 | 1403 ± 631 | 0.236 | -1.239 | 1467 ± 520 | 1002 ± 324 | 0.376 | 0.914 |

**Supplementary Table S2.** Accumulation of aromatic volatile compounds and camphene in undamaged (ctr) and *Melolontha melolontha*-damaged (herb) roots of *Populus trichocarpa* and *P. nigra.* Root material was extracted with hexane and analyzed using GC-MS/FID. Accumulation levels are displayed as means ± SE in µg $g^{-1}$ fresh weight (n = 8). *P*-values are based on the results from Student's t-tests or from Mann-Whitney Rank Sum Tests between control and herbivore treatments.

| | ***P. trichocarpa*** | | | | ***P. nigra*** | | | |
|---|---|---|---|---|---|---|---|---|
| | **ctr** | **herb** | ***P*-value** | **t-value/ *T-value*** | **ctr** | **herb** | ***P*-value** | **t-value/ T-value** |
| camphene | NA | NA | NA | NA | 0.0 ± 0.0 | 0.3 ± 0.1 | 0.002 | *40.00* |
| benzaldehyde | 7.9 ± 4.4 | 48.9 ± 6.6 | ≤0.001 | *37.00* | 0.1 ± 0.05 | 0.2 ± 0.03 | 0.373 | -0.920 |
| benzyl alcohol | 11.9 ± 1.4 | 1.9 ± 1.9 | ≤0.001 | 5.298 | NA | NA | NA | NA |
| salicylaldehyde | 550.1 ± 135.4 | 1948.6 ± 209.5 | ≤0.001 | -7.408 | 33.4 ± 4.3 | 73.4 ± 8.9 | ≤0.001 | -4.538 |

**Supplementary Table S3:** Oligonucleotides used for isolation, qRT-PCR analysis and site-directed mutagenesis of *TPS* genes.

| Name | Sequence | Usage |
|---|---|---|
| PtTPS16-PtTPS21-fwd | CACCATGGTAGCGACCGAAACTTG | cloning of *PtTPS16/21* |
| PtTPS16-rev | CTATATATCTTGGAGAGGAATGG | cloning of *PtTPS16* |
| PtTPS21-rev | GATTTATCAAGAAAACTTAAC | cloning of *PtTPS21* |
| PnTPS4-fwd | ATGGTAGGTCTCAGCGCGTTGCCACCGAAGCTGCTGGT | cloning of *PnTPS4* |
| PnTPS4-rev | ATGGTAGGTCTCATATCATAAAGGCTTAATAAGTAAGGATTTCAC | cloning of *PnTPS4* |
| PnTPS1-PtTPS21-qRT-PCR-fwd | TATCGAAAAATGGCGACCAGGG | qRT-PCR |
| PnTPS1-PtTPS21-qRT-PCR-rev | GCAGTTGGTCCAATGCATTGAT | qRT-PCR |
| PtTPS16-qRT-PCR-fwd | TATCGAAAAATGGCGACCAGGA | qRT-PCR |
| PtTPS16-qRT-PCR-rev | GCAGTTGGTCCAATGCATTGAC | qRT-PCR |
| PnTPS4-qRT-PCR-fwd | CATATACAGCGCGAATCGAAAAGT | qRT-PCR |
| PnTPS4-qRT-PCR-rev | AGATAACCCAAGTCTTTGCAAGGC | qRT-PCR |
| PtTPS16-I335V-fwd | ATGGCGACCAGGGTTACTGCTCTAATAACAGCATTAGAT | site-directed mutagenesis |
| PtTPS16-I335V-rev | ATCTAATGCTGTTATTAGAGCAGTAACCCTGGTCGCCAT | site-directed mutagenesis |
| PtTPS16-T336N-fwd | ATGGCGACCAGGATTAATGCTCTAATAACAGCATTAGAT | site-directed mutagenesis |
| PtTPS16-T336N-rev | ATCTAATGCTGTTATTAGAGCATTAATCCTGGTCGCCAT | site-directed mutagenesis |
| PtTPS16-V444I-fwd | GATAATGCTTGGTTGTCAATTTCCGGACAAGTCATACTA | site-directed mutagenesis |
| PtTPS16-V444I-rev | TAGTATGACTTGTCCGGAAATTGACAACCAAGCATTATC | site-directed mutagenesis |
| PtTPS16-V483L-fwd | CGTCACTCGTCAACGATTTTGCGACTCGCAGATGACCTA | site-directed mutagenesis |
| PtTPS16-V483L-rev | TAGGTCATCTGCGAGTCGCAAAATCGTTGACGAGTGACG | site-directed mutagenesis |
| PtTPS16-I335V-T336N-fwd | ATGGCGACCAGGGTTAATGCTCTAATAACAGCATTAGAT | site-directed mutagenesis |
| PtTPS16-I335V-T336N-rev | ATCTAATGCTGTTATTAGAGCATTAACCCTGGTCGCCAT | site-directed mutagenesis |
